# Lifespan Developmental Analysis of Health Problems and Problem Drinking Across White and Underrepresented Adults

**DOI:** 10.1093/geroni/igab046.3708

**Published:** 2021-12-17

**Authors:** Willard Boyd, Yimei Li, Mohammed Ahmed, Dania Mohammed, Thomas Kwan, Matthew Lee

**Affiliations:** 1 Rutgers The State University of NJ, Weehawken, New Jersey, United States; 2 Rutgers The State University of NJ, Piscataway, New Jersey, United States; 3 Rutgers The State University of New Jersey, Rutgers Center of Alcohol & Substance Use Studies, New Jersey, United States; 4 Rutgers Center of Alcohol & Substance Use Studies, Rutgers Center of Alcohol & Substance Use Studies, New Jersey, United States; 5 Rutgers University, Manalapan, New Jersey, United States; 6 Rutgers University, Piscataway, New Jersey, United States

## Abstract

Research has illustrated potential health benefits from moderate drinking, but also health risks from excessive drinking. Few studies have contrasted drinking effects on health across different periods of the lifespan, and how such contrasts may vary across sociodemographic subpopulations. In this study, we investigated underrepresented racial and ethnic group status as a moderator of drinking effects on health across the lifespan. Analyses used data from two waves of a large U.S.-representative sample. We estimated a series of 3*3 between-persons ANOVAs testing effects of Wave-1 drinking group (abstainer, moderate drinkers, and excessive drinkers), age (young adulthood, midlife, and older adulthood), and drinking-group-by-age interactions in White versus underrepresented status. The outcome variable was Wave-2 hypertension (controlling for Wave-1 hypertension). In the older-adult White group, results reflected the familiar “j-shaped” curve of alcohol effects on health. Specifically, abstainers experienced higher hypertension than moderate drinkers (with marginal significance: p=.054), and excessive drinkers experienced higher hypertension than moderate drinkers (p= .002). In contrast, among underrepresented older adults, hypertension levels did not vary significantly by drinking group. Graphical results clarified that the lack of drinking effects among underrepresented older adults reflected that they had similarly elevated hypertension across all three drinking groups, whereas the White older adults only had comparably elevated hypertension in the excessive-drinker group. These findings suggest that the positive health effects of moderate drinking apply primarily to White older adults. Our poster will discuss potential explanations for the apparent lack of health benefits of safe-drinking practices among underrepresented older adults.

